# Tuftsin: A Natural Molecule Against SARS-CoV-2 Infection

**DOI:** 10.3389/fmolb.2022.859162

**Published:** 2022-03-23

**Authors:** Jiahao Huang, Jing Wang, Yan Li, Ziyuan Wang, Ming Chu, Yuedan Wang

**Affiliations:** ^1^ Department of Immunology, School of Basic Medical Sciences, Peking University. NHC Key Laboratory of Medical Immunology(Peking University), Beijing, China; ^2^ State Key Laboratory of Natural and Biomimetic Drugs, School of Pharmaceutical Sciences, Peking University, Beijing, China

**Keywords:** tuftsin, SARS-CoV-2, ACE2, NRP1, natural peptide

## Abstract

Coronavirus disease 2019 (COVID-19) continuously progresses despite the application of a variety of vaccines. Therefore, it is still imperative to find effective ways for treating COVID-19. Recent studies indicate that NRP1, an important receptor of the natural peptide tuftsin (released from IgG), facilitates SARS-CoV-2 infection. Here, we found 91 overlapping genes between tuftsin targets and COVID-19-associated genes. We have demonstrated that tuftsin could also target ACE2 and exert some immune-related functions. Molecular docking results revealed that tustin could combine with ACE2 and NRP1 in stable structures, and their interacted regions cover the binding surfaces of S1-protein with the two receptors. Using surface plasmon resonance (SPR) analysis, we confirmed that tuftsin can bind ACE2 and NRP1 directly. Importantly, using SPR-based competition assay we have shown here that tuftsin effectively prevented the binding of SARS-CoV-2 S1-protein to ACE2. Collectively, these data suggest that tuftsin is an attractive therapeutic candidate against COVID-19 and can be considered for translational as well as clinical studies.

## Introduction

Coronavirus disease 2019 (COVID-19) caused by severe acute respiratory syndrome coronavirus 2 (SARS-CoV-2) results in high morbidity and mortality (Zhu et al., 2020; Dai and Gao, 2021). It is known that the spike (S) protein binding to angiotensin-converting enzyme 2 (ACE2) is the core mechanism of SARS-CoV-2 infecting host cells. COVID-19 vaccines have been approved for human use in most countries in the world. However, the concentration of neutralizing antibodies induced by vaccines still needs to be investigated in humans. Another effective way is the neutralizing monoclonal antibodies (mAbs) at present (Renn et al., 2020). However, the production of safe and effective mAbs is complicated, and the duration of effective protection remains to be determined (Su et al., 2021; Taylor et al., 2021). Moreover, continuous mutations of SARS-CoV-2 during the pandemic may cause coronavirus to escape from antibody recognition and reduce the neutralizing activity of mAbs (Du et al., 2021). Therefore, the discovery of a broad-spectrum and effective method for preventing and treating COVID-19 is crucial.

Along with ACE2, neuropilin 1 (NRP1) is another important host factor for SARS-CoV-2 infection (Daly et al., 2020). It has been reported that NRP1 facilitates the entry of SARS-CoV-2 into cells in the presence of ACE2 (Cantuti-Castelvetri et al., 2020). It is worth noting that NRP1 is an important receptor of tuftsin (Vander Kooi et al., 2007; von Wronski et al., 2006). Tuftsin, a natural phagocytosis-stimulating peptide, was found by Najjar and Nishioka (1970). Tuftsin is released from the Fc fragment of IgG by enzyme cleavage of an endocarboxy-peptidase in the spleen and a leukokininase on the outer membrane of neutrophilic leukocytes (Najjar and Nishioka, 1970; Corazza et al., 1991). Furthermore, tuftsin is a tetrapeptide that consists of Thr-Lys-Pro-Arg, located at amino acid residues 289 to 292 of the heavy chain of IgG (Kozlovskaya et al., 2003). Tuftsin has a broad spectrum of activities mainly associated with immune functions and exerts effects on phagocytic cells, especially macrophages. The function of tuftsin includes cell phagocytosis, motility, immunogenic response, and bactericidal and tumoricidal activities (Najjar, 1983; Fridkin and Najjar, 1989). Spleen is the only organ that produces tuftsin in mammals, and splenic defect caused by spleen injury or diseases often reduces the activity of tuftsin (Chu et al., 2015; Chapman et al., 2022). And it is reported that tuftsin activity is significantly lower in patients with AIDS, cirrhosis, intestinal failure and some infectious diseases than the normal individuals (Corazza et al., 1991; Zoli et al., 1998; Trevisani et al., 2002). Moreover, it was demonstrated that tuftsin has stability and low toxicity *in vitro* and *in vivo* (Amoscato et al., 1983; Fridkin and Najjar, 1989; Siemion and Kluczyk, 1999). It is worth noting that the mutant sequence of tuftsin turns to inactive or inhibitory analogs (Blumenstein et al., 1979; Najjar, 1981). As a natural immune stimulating peptide, tuftsin is an attractive candidate for immunotherapy. We hypothesized that tuftsin could inhibit SARS-CoV-2 infection by interacting with human receptors of coronavirus. The experiments were subsequently performed to test our concepts. In this study, the targets of tuftsin were collected from articles and bioinformatics prediction and the disease-related genes of COVID-19 were mined from database. Next, we selected the intersected proteins for further analyses. According to the theoretical basis and the enrichment results that tuftsin could target ACE2 and NRP1 in the COVID-19 pathway, the possible interactions of tuftsin with the two SARS-CoV-2 entry receptors were determined by molecular docking analyses. Tuftsin is further confirmed to bind the two SARS-CoV-2 entry receptors directly and significantly impair the binding of viral S1-protein to its human ACE2 receptor by using the surface plasmon resonance analyses and competition assays, shedding light on tuftsin-based new drug discovery against COVID-19.

## Materials and Methods

### Compound Profiling and Disease-Related Gene Identification

The structure of tuftsin was found in PubChem (https://pubchem.ncbi.nlm.nih.gov/). The 3D structure of tuftsin was built using Chem3D. Afterward, the target proteins corresponding to tuftsin screened from the Pharmmapper database and PubMed database were standardized in UniProt (http://www.uniprot.org/). Finally, Cytoscape 3.8.2 was used to determine the drug-target network. COVID-19-related genes were mined from the GeneCards database. All of the disease gene targets were normalized with R software using the Bioconductor package when redundancy was deleted (Yu et al., 2012).

### Network Establishment

Screening for drug-disease crossover genes was performed. Based on previous steps, two sets of target lists were prepared: drug targets and disease-related genes. The crossover genes were filtered with R software using the Venn Diagram package. The STRING 11.5 database (http://string-db.org/) was used to analyse the intersecting protein–protein interactions (PPIs), and the common targets were counted with R software.

### Enrichment Analysis

The proteins with overlapping expression patterns were evaluated by bioinformatics annotation with R software using the Bioconductor package, including a panther classification system (http://www.pantherdb.org/), a gene ontology (GO) annotation database website (http://www.geneontology.org), and Kyoto Encyclopedia of Genes and Genomes (KEGG) pathway enrichment analysis (http://www.genome.jp/kegg/). A *p* < 0.05 was considered statistically significant.

### Molecular Docking Analysis

The flexible docking process between tuftsin and target proteins was conducted by softwere Discovery Studio 2021 (DS). Briefly, the crystallographic structures of human ACE2 (PDB ID: 1R42) and human NRP1 (PDB ID: 2QQ1) with high resolution were prepared using the Prepare Protein and Minimization module of DS. The active binding site of each protein was defined based on the most representative features of the SARS-CoV-2 interface. Tuftsin was docked into the active binding site of ACE2 and NRP1 using the molecular docking module in DTS.

### Surface Plasmon Resonance Analysis

The recombinant human ACE2 protein (Novoprotein, Beijing, China) and recombinant human NRP1 protein were used for surface plasmon resonance (SPR) analysis using a Biacore T200 instrument (Biacore, Uppsala, Sweden). Each target was immobilized onto flow cells in a CM5 sensor chip (GE Healthcare) via the amine-coupling method. Briefly, ACE2 and NRP1 were diluted in 10 mM pH 4.5 acetate to 20 μg/ml. Then, the protein solutions were injected individually on the carboxyl-modified sensor surface to form amine bonds. Both ACE2 and NRP1 immobilized levels were approximately 10,000 RU. Binding analyses were carried out at 25°C and a flow rate of 30 µl/min. Tuftsin diluted in running buffer (1×PBS, 0.05% Tween 20 and 5% dimethyl sulfoxide, pH 7.4) was run over each target at gradient concentrations. An empty flow cell without any immobilized protein was used as a deducted reference. The binding curves were analysed using the steady state affinity model supplied with Biacore Evaluation Software (GE Healthcare).

### Competition Binding Experiment

For the competition binding experiment, the SARS-CoV-2 S1 protein was immobilized on the CM5 sensor chip via the amine-coupling method. 5 nM ACE2 was injected for negative control. Tuftsin was diluted into a series of solutions with gradient concentrations and fixed with 5 nM ACE2, and then the solutions were injected into the chip. The blocking efficacy was evaluated by comparison of response units with and without tuftsin incubation.

### Statistical Analysis

Partial results were analysed using chi-square test with SPSS software and R 4.1.0.

## Results

### Bioinformatics Analyses Revealed the Connection Between Tuftsin and COVID-19

**FIGURE 1:**
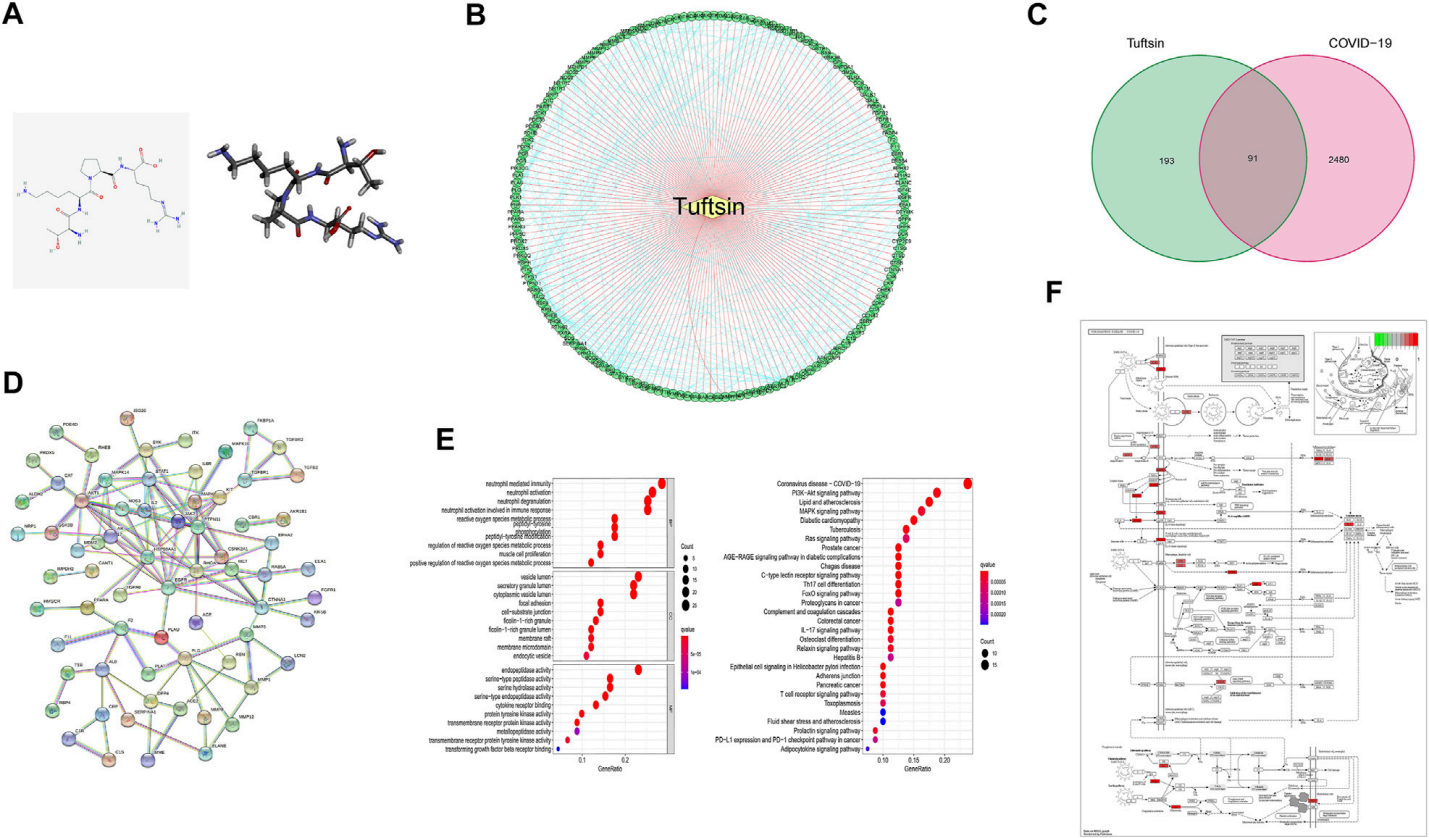
The connection between tuftsin and COVID-19. **(A)** (Left) The 2D chemical structure of tuftsin downloaded from the PubChem database. (Right) The 3D chemical structure of tuftsin established by software based on the 2D structure. **(B)** The “drug-target” network of tuftsin. Red links represent the interactions between tuftsin and target nodes. Each node is a protein target. Green points represent the targeted proteins in humans. Blue links represent the interactions between the targets. **(C)** A Venn diagram of tuftsin and COVID-19 cotargeted genes. **(D)** Protein–protein interaction (PPI) network of the intersected targets. The interactions with a high confidence of 0.95. **(E)** (Left) Gene ontology enrichment results in bubble plot. (Right) The KEGG enrichment results in bubble plot. **(F)** Detailed targets of tuftsin in the COVID-19 pathway. Red points represent the tuftsin targets. The intensity of the color represents the possibility of tuftsin targeting. Deeper color indicates higher possibility.

The 2D structure of tuftsin was obtained from the PubChem database (Compound CID: 156080), and the most stable 3D structure was built based on the 2D structure through a molecular simulation assay (Figure 1A). In addition to the reported receptors of tuftsin, the potential targets of tuftsin in human body were predicted through the PharmMapper database. As a result, 284 targets of tuftsin were selected (Figure 1B and Supplementary Data S1), and 2,572 disease-associated genes of COVID-19 were excavated from the GeneCards database (Supplementary Data S2). It is surprised to find that there exists 91 intersecting proteins between tuftsin targets and COVID-19-associated gene-coded proteins through intersection analysis, and the overlapping proteins account for nearly one-third of tuftsin targets (Figure 1C). The protein–protein interaction network of the overlapping proteins was established, and the result indicated that JAK2, STAT1 and AKT1 are core molecules in the network (Figure 1D). The 91 intersecting genes were further studied by enrichment analysis. GO and KEGG annotation revealed that the expressed tuftsin-COVID-19 crossover proteins are mainly associated with immune functions such as neutrophil activation, neutrophil-mediated immunity and cytokine receptor binding, and the COVID-19 pathway is most significantly enriched. In addition, many target genes were strongly associated with some immune pathways, such as Th17 cell differentiation, the IL-17 signaling pathway and the immune checkpoint pathway (Figure 1E). In the COVID-19 pathway, the SARS-CoV-2 receptors ACE2 and NRP1, some immune molecules such as IL-2, STAT1, and some complement molecules are the targets of tuftsin (Figure 1F). These results suggest that tuftsin is a promising candidate against COVID-19 owing to its multifaceted pharmacological activities.

### The Interaction of Tuftsin With ACE2 and NRP1 Analysed by Molecular Docking

**FIGURE 2:**
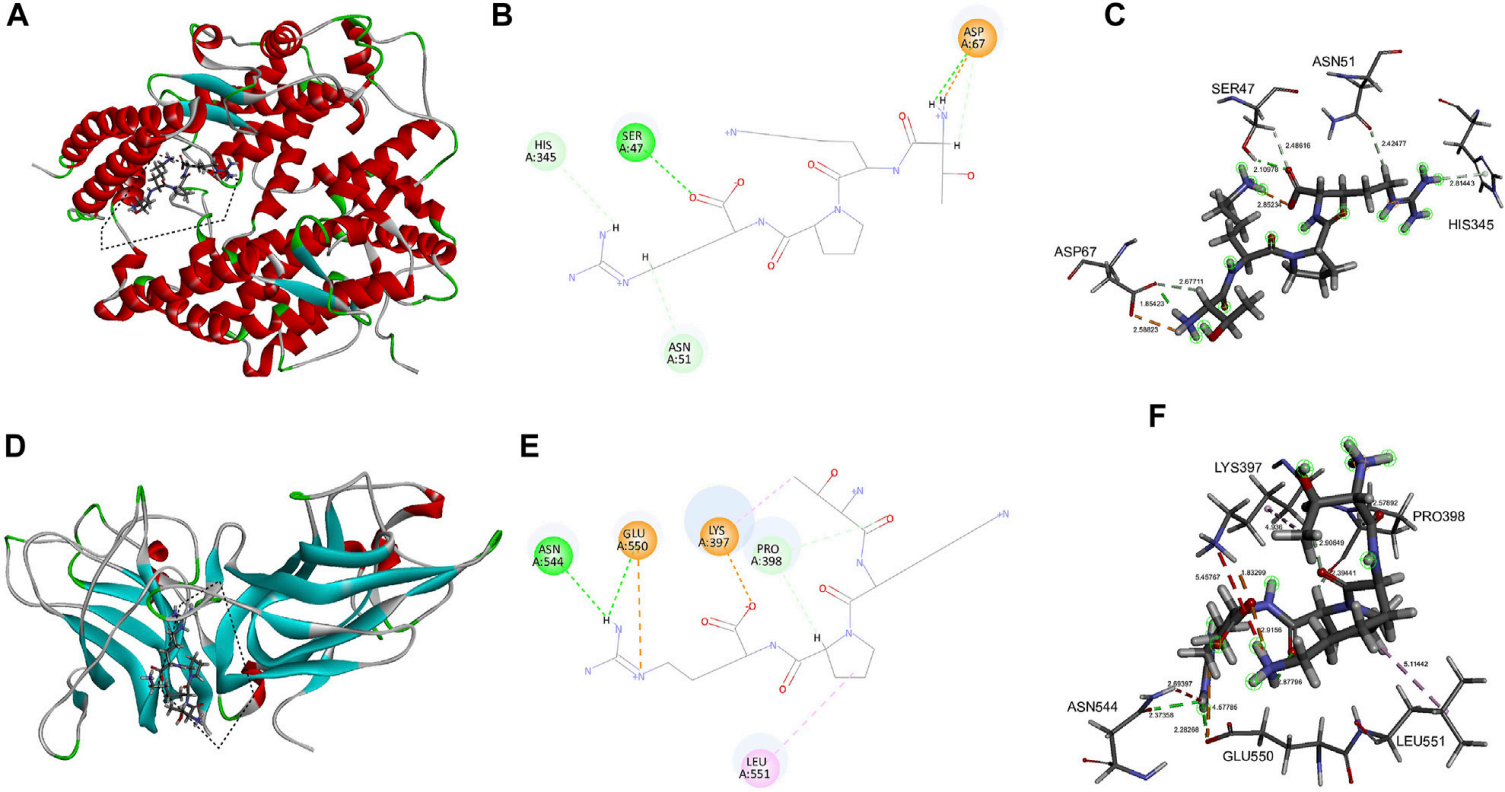
Molecular interaction of tuftsin with ACE2 and NRP1. **(A)** The binding pattern of tuftsin with ACE2. Binding area was circled by black dotted line. Secondary structural elements are depicted as ribbons (coils, α-helices, arrows, β-sheets). Color is based on secondary structures (α-helices, red; β-sheets, skyblue; loops, green). **(B)** Molecular interaction schemes of tuftsin with the relative residues of ACE2. Green lines represent conventional hydrogen bonds; light green lines represent carbon-hydrogen bonds; orange lines represent salt bridges; and pink lines represent alkyl bonds. **(C)** The bond lengths of intermolecular interactions between tuftsin and ACE2. The bond length was indicated by the dotted line between the interacted molecules. The bound amino acid residues are shown in stick representation. **(D)** The binding pattern of tuftsin with NRP1. Binding area was circled by black dotted line. **(E)** Molecular interaction schemes of tuftsin with the relative residues of NRP1. **(F)** The bond lengths of intermolecular interactions between tuftsin and NRP1. Other interpretations are the same as above.

Having proved by the above pharmacology analysis of tuftsin, it is possible that ACE2 is a target of tuftsin. Therefore, the potential binding sites and binding affinity of tuftsin with the SARS-CoV-2 receptors ACE2 and NRP1 were further determined by molecular docking analysis. The interaction interface of SARS-CoV-2 S1 receptor-binding domain (RBD) with ACE2 was defined as the active sites of ACE2. These interface sites in ACE2 include Q24, M82, N330, and R393 residues, which are mainly located in the N-terminal peptidase domain of ACE2 (Lan et al., 2020). The docking region of ACE2 is a sphere containing the defined ACE2 active sites (Supplementary Figure S1A). The docking results showed that the affinity of tuftsin with ACE2 was −6.9 kcal/mol, demonstrating that they could combine spontaneously (Figure 2A). Furthermore, tuftsin could form strong hydrogen bonds to Ser47 and Asp67, hydrogen bonds to His345, Asp67 and Asn51, and salt bridges to Asp67 residues of ACE2 (Figure 2B). In addition, the bond lengths of the interacted sites are that 2.110 Å of Ser47, 1.854 Å of Asp67, 2.814 Å of His345 and 2.425 Å of Asn51 (Figure 2C). It is worth mentioning that the binding sites were adjacent to the interactional sites of S1-RBD and ACE2 (Lan et al., 2020), indicating that tuftsin could inhibit S1 binding to ACE2 by covering their binding sites. It is reported that the extracellular b1b2 domain of NRP1 combines with S1 CendR peptides (Plein et al., 2014; Daly et al., 2020), therefore the b1b2 domain of NRP1 was prepared for docking. The interactional sites of S1-RBD with b1b2 proteins were defined as the active sites of NRP1 b1b2 domain, including D320, E348, Y353 residues and so on (Daly et al., 2020). Subsequently, the docking region was a sphere containing these defined active sites in the b1b2 domain of NRP1 (Supplementary Figure S1B). The docking results showed that tuftsin and NRP1 b1b2 domain have a high binding affinity of −8.1 kcal/mol. In addition, tuftsin fits solidly into a binding pocket on NRP1 b1b2 domain (Figure 2D). Furthermore, tuftsin could form a salt bridge to Lys397 and a carbon-hydrogen bond to Pro398 residues of NRP1 (Figure 2E), which are near the interactional sites of S1-RBD with NRP1 b1b2 domain. The shortest bond lengths of these interacted sites are that 1.833 Å of Lys397 and 2.394 Å of Pro398 (Figure 2F). Moreover, the binding region of tuftsin and NRP1 overlapped with the binding area of NRP1 with S1-RBD in space. Collectively, these results demonstrated that tuftsin could bind ACE2 and NRP1, and inhibit the SARS-CoV-2 S1 binding to ACE2 and NRP1 by covering their interactional sites.

### Tuftsin Binds ACE2 and NPR1 Directly, as Confirmed by Surface Plasmon Resonance (SPR) Analyses

**FIGURE 3:**
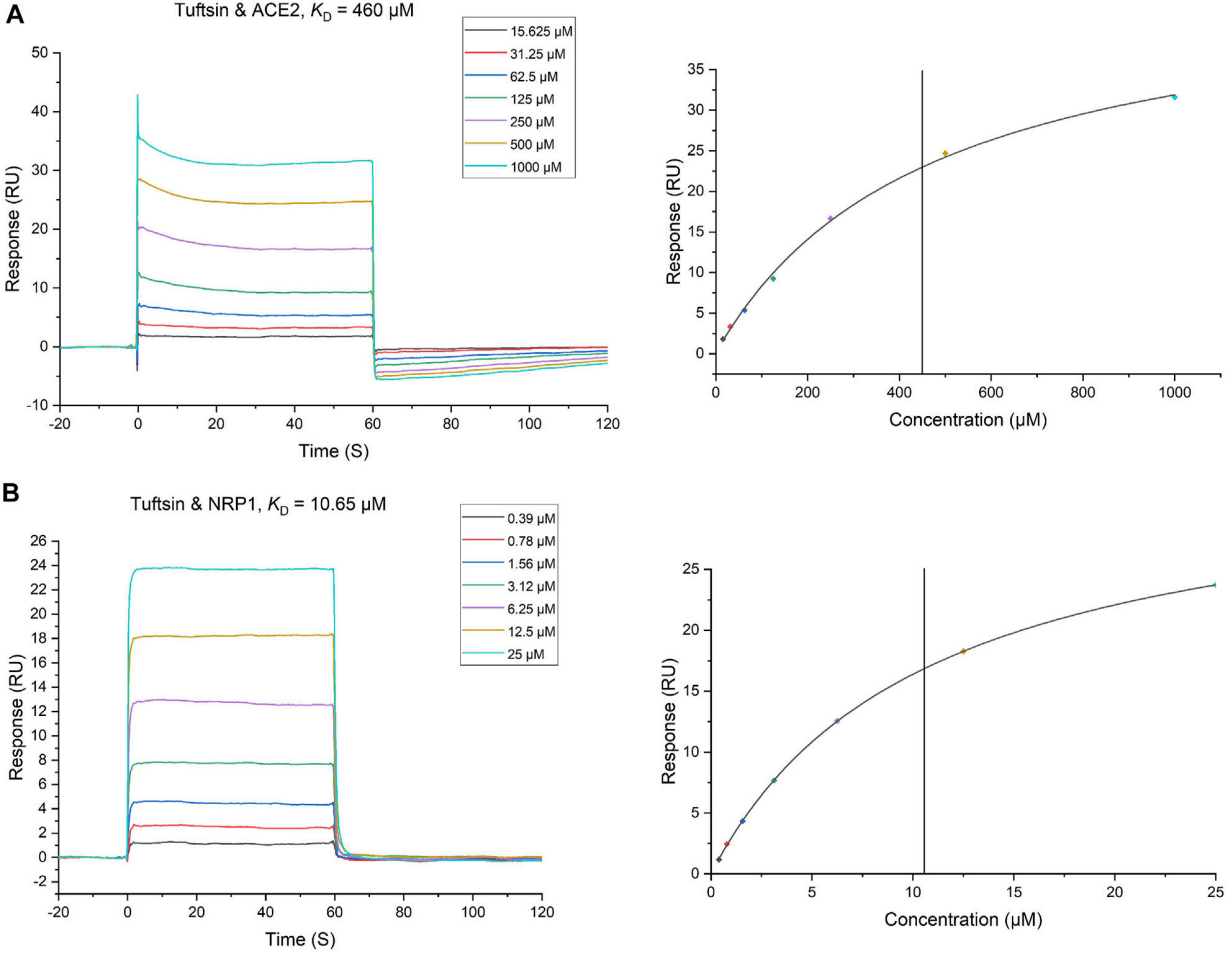
The binding of tuftsin to ACE2 and NRP1 was determined by SPR assay. **(A)** (Left) Binding curves of tuftsin with ACE2. The *K*_D_ of the ACE2 protein with a series of concentrations of tuftsin was calculated by using a 1:1 binding model. Data are presented as response units (RU) over time (S). (Right) The fitting carve of tuftsin with ACE2. **(B)** (Left) Binding curves of tuftsin with NRP1. The *K*_D_ of the NRP1 protein with a series of concentrations of tuftsin was calculated by using a 1:1 binding model. Other interpretations are the same as above. (Right) The fitting carve of tuftsin with NRP1.

The interactions of tuftsin with ACE2 and NRP1 were further evaluated by real-time biomolecular interaction analysis with SPR. The steady state affinity of the binding reaction were determined by injecting different concentrations of tuftsin over recombinant human ACE2 immobilized on one half of the chip surface, and over recombinant human NRP1 immobilized on another half of the chip surface. The results showed that tuftsin can bind ACE2 with an equilibrium dissociation constant (*K*_D_) of 460 μmol/L (Figure 3A). Moreover, the *K*_D_ fitting curve of tuftsin with ACE2 is gradually stable when the concentrations of tuftsin increased gradually, indicating that the interaction of tuftsin and ACE2 is specific (Figure 3A). Tuftsin can also bind NRP1 strongly with a higher binding affinity with *K*_D_ value calculated as 10.65 μmol/L (Figure 3B). The *K*_D_ fitting curve of tuftsin with NRP1 is gradually stable when the concentrations of tuftsin increased gradually, conforming to the characteristic of specific binding (Figure 3B). As SPR is the gold standard for detecting drug-target interactions, these results demonstrate that tuftsin binds ACE2 and NRP1 directly and specifically with ideal affinities, and accord with the accuracy of the previous results of bioinformatics analyses and molecular docking assays.

### Tuftsin Impairs the Binding of SARS-CoV-2 S1 to ACE2

**FIGURE 4:**
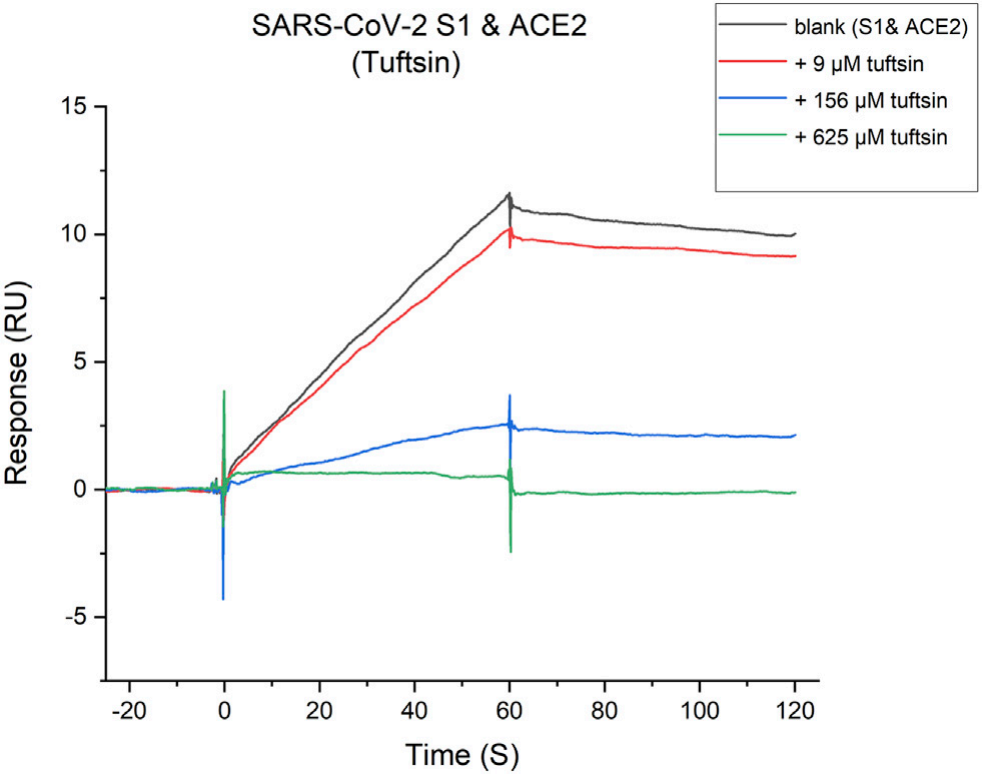
Tuftsin inhibits the SARS-CoV-2 S1 binding to ACE2. The binding activity of SARA-CoV-2 S1-protein to ACE2 in the presence of increasing concentrations of tuftsin. The gray “blank” line represents the binding response of S1-protein with ACE2 alone for negative control. Then, multiple concentrations of tuftsin were added based on the “blank” for comparison. Intensive concentrations of tuftin showed enhanced inhibitory effects.

Whether tuftsin could affect the binding of S1 protein with ACE2 was further determined by SPR-based competition assay. The binding affinity of the S1 protein with ACE2 was firstly determined by SPR assay, which unsurprisingly showed a high affinity. A suitable concentration ACE2 solution was injected over the immobilized SARS-CoV-2 S1 protein as a control. A series of gradient concentrations of tuftsin solutions containing equal concentrations of ACE2 were injected over the immobilized SARS-CoV-2 S1 protein for comparison. As a result, 9 μmol/L tuftsin has a mild inhibitory effect. It is worth noting that the addition of 156 μmol/L tuftsin significantly attenuates the response signal about two-thirds compared to that of ACE2 alone over the immobilized S1. Notably, a substantial decrease in the response signal was observed with increasing concentrations of tuftsin. The response single is declined approximately to zero when the added concentration of tuftsin was 625 μmol/L. This result indicates that the interaction between S1 and ACE2 is completely abrogated in the presence of 625 μmol/L tuftsin (Figure 4). The experiment was repeated three times independently. In conclusion, the competition binding experiment revealed that tuftsin effectively impairs the binding of SARS-CoV-2 S1 to ACE2 in a dose-dependent manner.

## Discussion

Vaccination is widely used to prevent COVID-19 at present (Forni et al., 2021). The costs of production of vaccines and neutralizing antibodies for COVID-19 are relatively high, and it is reported that the effectiveness of the SARS-CoV-2 vaccines has declined significantly since 2021 (Cohn et al., 2021). Nonetheless, some collaborative methods can be applied to work with vaccines. It is demonstrated that peptides could be a promising approach to combat COVID-19 (Schütz et al., 2020). In this study, a human natural peptide holds great potential against SARS-CoV-2 virus is reported the first time. It is found that tuftsin could target many disease-related proteins of COVID-19 by intersection analysis. The COVID-19 pathway and some immune-related functions are highly relevant to these intersecting targets indicated by enrichment analysis. ACE2 and NRP1 are outstanding in these intersecting targets. ACE2 is a major receptor of SARS coronavirus’ spike (S) protein, and facilitates the subsequent membrane fusion (Jackson et al., 2022). NRP1 was confirmed to bind with S1 CendR motif and the furin-cleaved substrates, and significantly potentiates SARS-CoV-2 infectivity (Cantuti-Castelvetri et al., 2020; Daly et al., 2020). Subsequently, the combined constructions and binding affinity of tuftsin with ACE2 and NRP1 were determined by molecular docking analysis. As a result, tuftsin could combine with ACE2 and NRP1 in stable structures, with binding free energy of −6.9 kcal/mol and -8.1 kcal/mol, respectively. These interacted molecules are mainly linked with hydrogen bonds, carbon-hydrogen bonds and salt bridges. More importantly, it was found that the bond energy of these binding forces are high with low bond lengths ranging from 1.8 Å to 2.9 Å, indicating the complexes of tuftsin with ACE2 and NRP1 are stable. The binding sites of tuftsin with ACE2 and NRP1 were next to the binding sites of SARS-CoV-2 S1-RBD with the two receptors, such as Ser47, His345, Asp67 residues of ACE2 and Lys397, Pro398 residues of NRP1. These results indicate tuftsin could inhibit SARS-CoV-2 binding to ACE2 and NRP1 by covering their interacted regions competitively.

To further confirm the inhibiting properties of tustsin, we conducted the surface plasmon resonance (SPR) analysis. Tuftsin showes a moderate binding affinity to ACE2 with a *K*_D_ at 460 μmol/L, and showes an even higher binding affinity to NRP1 with a *K*_D_ value of 10.65 μmol/L, which are actually very good values for an unmodified natural peptide. More importantly, it was found that the viral S1 protein binding to ACE2 receptor was almost abrogated in the presence of tuftsin at 625 μM. These indicate tuftsin plays important roles in inhibiting SARS-CoV-2 by impairing the binding of S1 protein with ACE2 significantly, which is an important way of preventing virus infection. Tuftsin as a natural and unmodified tetrapeptide which exists in human bodies, originates from a special fraction of the parent carrier IgG (Siebert et al., 2017). Therefore, tuftsin may have lower toxicity and side effects than other synthetic and exogenous drugs (Catane et al., 1983). It is reported that approved drugs which main ingredient is tuftsin have satisfactory clinical efficacy at present, in addition, tuftsin incorporates specific molecules such as tuftsin-phosphorylcholine and tuftsin-bearing liposomes, showing greater immunogenicity and specific targeting (Shakya et al., 2012; Croci et al., 2019; Khan, 2021). Tuftsin can be produced on a large scale at low cost (Siebert et al., 2017), which allows tuftsin to be widely applied for against COVID-19. These It has been reported that the amount of IgG induced by vaccines is mainly focused on the lower respiratory tract (Krammer, 2020). Therefore, tuftsin could be applied as spray form to protect the upper respiratory which suffers coronavirus infection. It is worth noting that there exists a group of asymptomatic carriers or mild symptoms’ individuals during the pandemic (Schijns and Lavelle, 2020; Boyton and Altmann, 2021). As tuftsin has anti-infection and anti-inflammatory properties (Blok-Perkowska et al., 1984; Thompson et al., 2018), it is possible that asymptomatic individuals have higher content of tuftsin.

In conclusion, a natural peptide, tuftsin, shows high potency of treating COVID-19 along with the immunomodulation effect, and exhibits satisfactory ACE2 and NRP1-targeting ability. More importantly, tuftsin is demonstrated to impair the interaction between S1-protein and ACE2. This study provided direct biophysics evidences associated with molecular mechanisms of possible clinical use of tuftsin for prevention and treatment of COVID-19. In addition, this study demonstrated the utility of human natural peptide has good potency in treating COVID-19. Therefore, tuftsin is a promising anti-SARS-CoV-2 drug candidate.

## Data Availability

The original contributions presented in the study are included in the article/Supplementary Material, further inquiries can be directed to the corresponding authors.

## References

[B1] Amoscato A. A. Davies P. J. Babcock G. F. Nishioka K. (1983). Receptor-mediated Internalization of Tuftsin by Human Polymorphonuclear Leukocytes. J. Reticuloendothel Soc. 34, 53–67. 6308252

[B2] Blok-Perkowska D. Muzalewski F. Konopińska D. (1984). Antibacterial Properties of Tuftsin and its Analogs. Antimicrob. Agents Chemother. 25, 134–136. 10.1128/aac.25.1.134 6703677 PMC185452

[B3] Blumenstein M. Layne P. P. Najjar V. A. (1979). Nuclear Magnetic Resonance Studies on the Structure of the Tetrapeptide Tuftsin L-Threonyl-L-Lysyl-L-Prolyl-L-Arginine, and its Pentapeptide Analog L-Threonyl-L-Lysyl-L-Prolyl-L-Prolyl-L-Arginine. Biochemistry 18, 5247–5253. 10.1021/bi00590a032 40597

[B4] Boyton R. J. Altmann D. M. (2021). The Immunology of Asymptomatic SARS-CoV-2 Infection: what Are the Key Questions? Nat. Rev. Immunol. 21 (12), 762–768. 34667307 10.1038/s41577-021-00631-x PMC8525456

[B5] Cantuti-Castelvetri L. Ojha R. Pedro L. D. Djannatian M. Franz J. Kuivanen S. (2020). Neuropilin-1 Facilitates SARS-CoV-2 Cell Entry and Infectivity. Science 370, 856–860. 10.1126/science.abd2985 33082293 PMC7857391

[B6] Catane R. Schlanger S. Weiss L. Penchas S. Fuks Z. Treves A. J. (1983). Toxicology and Antitumor Activity of Tuftsin. Ann. NY Acad. Sci. 419, 251–260. 10.1111/j.1749-6632.1983.tb37111.x 6370073

[B7] Chapman J. Bansal P. Goyal A. Azevedo A. M. (2022). Splenomegaly, StatPearlsCopyright © 2022. Treasure Island (FL): StatPearls Publishing LLC.

[B8] Chu H. Han W. Wang L. Xu Y. Jian F. Zhang W. (2015). Long-term Efficacy of Subtotal Splenectomy Due to portal Hypertension in Cirrhotic Patients. BMC Surg. 15, 89. 10.1186/s12893-015-0077-2 26205377 PMC4511991

[B9] Cohn B. A. Cirillo P. M. Murphy C. C. Krigbaum N. Y. Wallace A. W. (2021). SARS-CoV-2 Vaccine protection and Deaths Among US Veterans during 2021. Sci. 2021, eabm0620. 10.1126/science.abm0620 PMC9836205 34735261

[B10] Corazza G. R. Ginaldi L. Profeta V. Quaglino D. Zoli G. Gasbarrini G. (1991). Tuftsin Deficiency in AIDS. The Lancet 337, 12–13. 10.1016/0140-6736(91)93331-3 1670648

[B11] Croci S. Bonacini M. Muratore F. Caruso A. Fontana A. Boiardi L. (2019). The Therapeutic Potential of Tuftsin-Phosphorylcholine in Giant Cell Arteritis. J. Autoimmun. 98, 113–121. 10.1016/j.jaut.2019.01.002 30638709

[B12] Dai L. Gao G. F. (2021). Viral Targets for Vaccines against COVID-19. Nat. Rev. Immunol. 21, 73–82. 10.1038/s41577-020-00480-0 33340022 PMC7747004

[B13] Daly J. L. Simonetti B. Klein K. Chen K.-E. Williamson M. K. Antón-Plágaro C. (2020). Neuropilin-1 Is a Host Factor for SARS-CoV-2 Infection. Science 370, 861–865. 10.1126/science.abd3072 33082294 PMC7612957

[B14] Du L. Yang Y. Zhang X. (2021). Neutralizing Antibodies for the Prevention and Treatment of COVID-19. Cell Mol Immunol 18, 2293–2306. 10.1038/s41423-021-00752-2 34497376 PMC8424621

[B15] Forni G. Mantovani A. Mantovani A. (2021). COVID-19 Vaccines: where We Stand and Challenges Ahead. Cell Death Differ 28, 626–639. 10.1038/s41418-020-00720-9 33479399 PMC7818063

[B16] Fridkin M. Najjar V. A. (1989). Tuftsin: Its Chemistry, Biology, and Clinical Potentia. Crit. Rev. Biochem. Mol. Biol. 24, 1–40. 10.3109/10409238909082550 2667894

[B17] Jackson C. B. Farzan M. Chen B. Choe H. (2022). Mechanisms of SARS-CoV-2 Entry into Cells. Nat. Rev. Mol. Cel Biol 23, 3–20. 10.1038/s41580-021-00418-x PMC8491763 34611326

[B18] Khan M. A. (2021). Targeted Drug Delivery Using Tuftsin-Bearing Liposomes: Implications in the Treatment of Infectious Diseases and Tumors. Cdt 22, 770–778. 10.2174/1389450121999201125200756 33243117

[B19] Kozlovskaya M. M. Kozlovskii II Val'dman E. A. Seredenin S. B. (2003). Selank and Short Peptides of the Tuftsin Family in the Regulation of Adaptive Behavior in Stress. Neurosci. Behav. Physiol. 33, 853–860. 10.1023/a:1025988519919 14969422

[B20] Krammer F. (2020). SARS-CoV-2 Vaccines in Development. Nature 586, 516–527. 10.1038/s41586-020-2798-3 32967006

[B21] Lan J. Ge J. Yu J. Shan S. Zhou H. Fan S. (2020). Structure of the SARS-CoV-2 Spike Receptor-Binding Domain Bound to the ACE2 Receptor. Nature 581, 215–220. 10.1038/s41586-020-2180-5 32225176

[B22] Najjar V. A. (1981). Biochemical Aspects of Tuftsin Deficiency Syndrome. Med. Biol. 59, 134–138. 6895538

[B23] Najjar V. A. Nishioka K. (1970). 'Tuftsin': a Natural Phagocytosis Stimulating Peptide. Nature 228, 672–673. 10.1038/228672a0 4097539

[B24] Najjar V. A. (1983). Tuftsin, a Natural Activator of Phagocyte Cells: an Overview. Ann. NY Acad. Sci. 419, 1–11. 10.1111/j.1749-6632.1983.tb37086.x 6370072

[B25] Plein A. Fantin A. Ruhrberg C. (2014). Neuropilin Regulation of Angiogenesis, Arteriogenesis, and Vascular Permeability. Microcirculation 21, 315–323. 10.1111/micc.12124 24521511 PMC4230468

[B26] Renn A. Fu Y. Hu X. Hall M. D. Simeonov A. (2020). Fruitful Neutralizing Antibody Pipeline Brings Hope to Defeat SARS-Cov-2. Trends Pharmacol. Sci. 41, 815–829. 10.1016/j.tips.2020.07.004 32829936 PMC7572790

[B27] Schijns V. Lavelle E. C. (2020). Prevention and Treatment of COVID‐19 Disease by Controlled Modulation of Innate Immunity. Eur. J. Immunol. 50, 932–938. 10.1002/eji.202048693 32438473 PMC7280664

[B28] Schütz D. Ruiz-Blanco Y. B. Münch J. Kirchhoff F. Sanchez-Garcia E. Müller J. A. (2020). Peptide and Peptide-Based Inhibitors of SARS-CoV-2 Entry. Adv. Drug Deliv. Rev. 167, 47–65. 33189768 10.1016/j.addr.2020.11.007 PMC7665879

[B29] Shakya N. Sane S. A. Haq W. Gupta S. (2012). Augmentation of Antileishmanial Efficacy of Miltefosine in Combination with Tuftsin against Experimental Visceral Leishmaniasis. Parasitol. Res. 111, 563–570. 10.1007/s00436-012-2868-z 22392136

[B30] Siebert A. Gensicka-Kowalewska M. Cholewinski G. Dzierzbicka K. (2017). Tuftsin - Properties and Analogs. Curr. Med. Chem. 24, 3711–3727. 10.2174/0929867324666170725140826 28745220

[B31] Siemion I. Z. Kluczyk A. (1999). Tuftsin: on the 30-year Anniversary of Victor Najjar's Discovery. Peptides 20, 645–674. 10.1016/s0196-9781(99)00019-4 10465518

[B32] Su W. Sia S. F. Schmitz A. J. Bricker T. L. Starr T. N. Greaney A. J. (2021). Neutralizing Monoclonal Antibodies that Target the Spike Receptor Binding Domain Confer Fc Receptor-independent Protection against SARS-CoV-2 Infection in Syrian Hamsters. mBio 12, e0239521. 10.1128/mbio.02395-21 34517754 PMC8546861

[B33] Taylor P. C. Adams A. C. Hufford M. M. de la Torre I. Winthrop K. Gottlieb R. L. (2021). Neutralizing Monoclonal Antibodies for Treatment of COVID-19. Nat. Rev. Immunol. 21, 382–393. 10.1038/s41577-021-00542-x 33875867 PMC8054133

[B34] Thompson K. K. Nissen J. C. Pretory A. Tsirka S. E. (2018). Tuftsin Combines with Remyelinating Therapy and Improves Outcomes in Models of CNS Demyelinating Disease. Front. Immunol. 9, 2784. 10.3389/fimmu.2018.02784 30555470 PMC6283261

[B35] Trevisani F. Castelli E. Foschi F. G. Parazza M. Loggi E. Bertelli M. (2002). Impaired Tuftsin Activity in Cirrhosis: Relationship with Splenic Function and Clinical Outcome. Gut 50, 707–712. 10.1136/gut.50.5.707 11950821 PMC1773217

[B36] Vander Kooi C. W. Jusino M. A. Perman B. Neau D. B. Bellamy H. D. Leahy D. J. (2007). Structural Basis for Ligand and Heparin Binding to Neuropilin B Domains. Proc. Natl. Acad. Sci. U.S.A. 104, 6152–6157. 10.1073/pnas.0700043104 17405859 PMC1851056

[B37] von Wronski M. A. Raju N. Pillai R. Bogdan N. J. Marinelli E. R. Nanjappan P. (2006). Tuftsin Binds Neuropilin-1 through a Sequence Similar to that Encoded by Exon 8 of Vascular Endothelial Growth Factor. J. Biol. Chem. 281, 5702–5710. 10.1074/jbc.m511941200 16371354

[B38] Yu G. Wang L.-G. Han Y. He Q.-Y. (2012). clusterProfiler: an R Package for Comparing Biological Themes Among Gene Clusters. OMICS: A J. Integr. Biol. 16, 284–287. 10.1089/omi.2011.0118 PMC3339379 22455463

[B39] Zhu N. Zhang D. Wang W. Li X. Yang B. Song J. (2020). A Novel Coronavirus from Patients with Pneumonia in China, 2019. N. Engl. J. Med. 382, 727–733. 10.1056/nejmoa2001017 31978945 PMC7092803

[B40] Zoli G. Corazza G. R. Wood S. Bartoli R. Gasbarrini G. Farthing M. J. G. (1998). Impaired Splenic Function and Tuftsin Deficiency in Patients with Intestinal Failure on Long Term Intravenous Nutrition. Gut 43, 759–762. 10.1136/gut.43.6.759 9824601 PMC1727358

