# Supplementary material for: Tuftsin: A Natural Molecule Against SARS-CoV-2 Infection
Source: Front Mol Biosci. 2022 Mar 23;9:859162. doi: 10.3389/fmolb.2022.859162 (PMC8984176; doi:10.3389/fmolb.2022.859162)
Supplement: Supplementary file 1 [file DataSheet1.docx]

Supplementary Material

# 1.Supplementary Data

Data S1.

The 284 tuftsin targets were collected from published and predicted results.


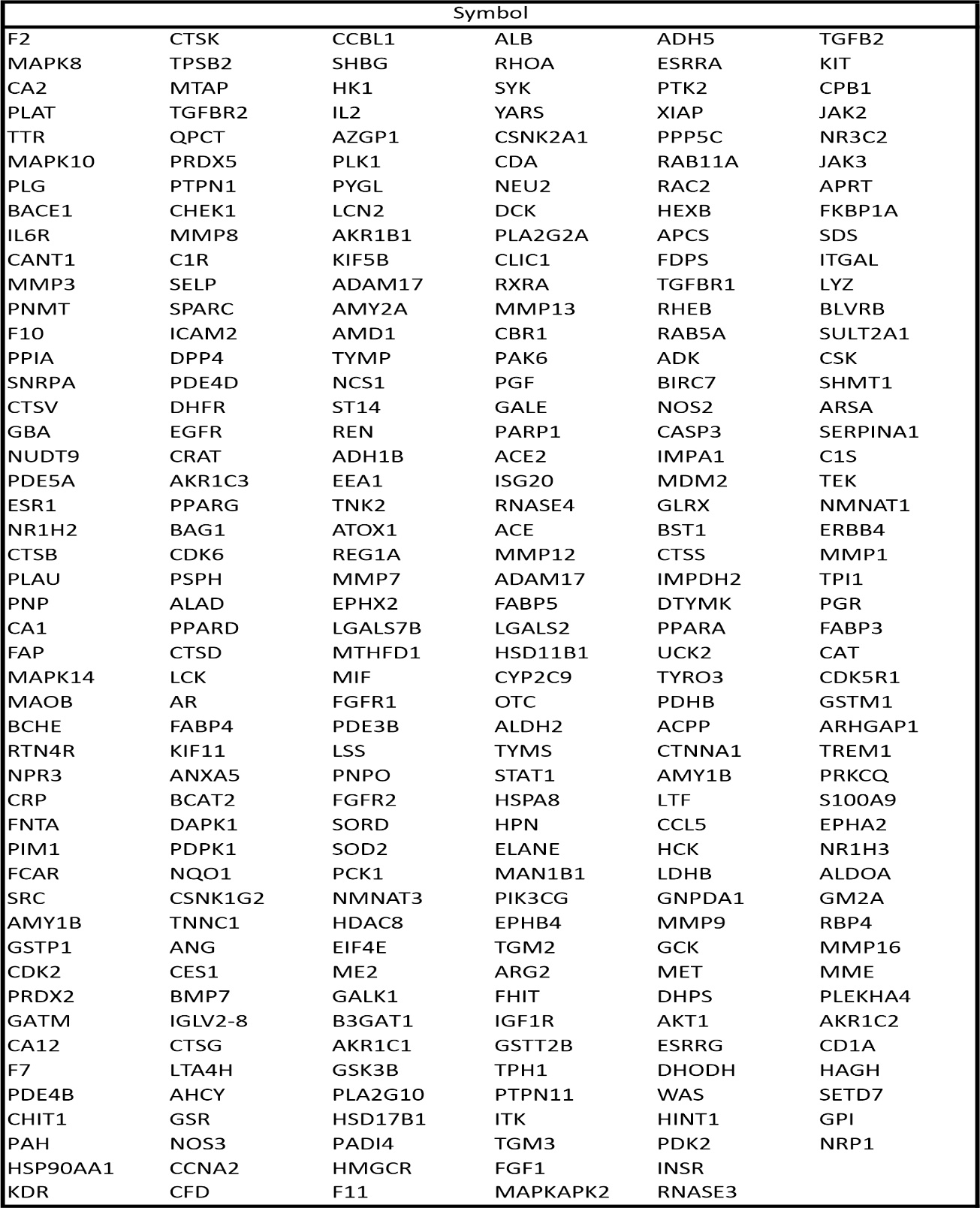


**Data S2.**

The 2572 disease-related genes of COVID-19 were collected from GeneCards.


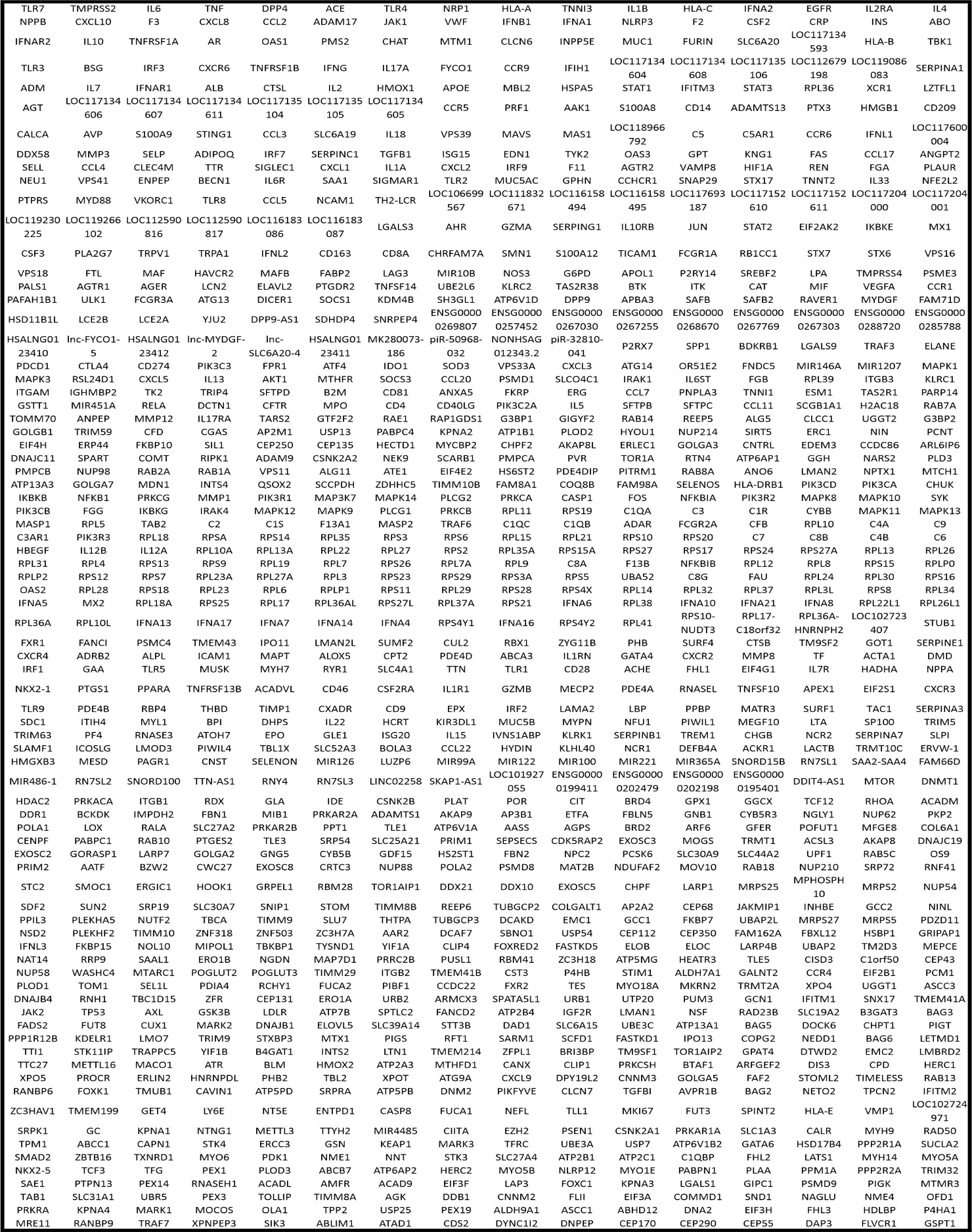


#
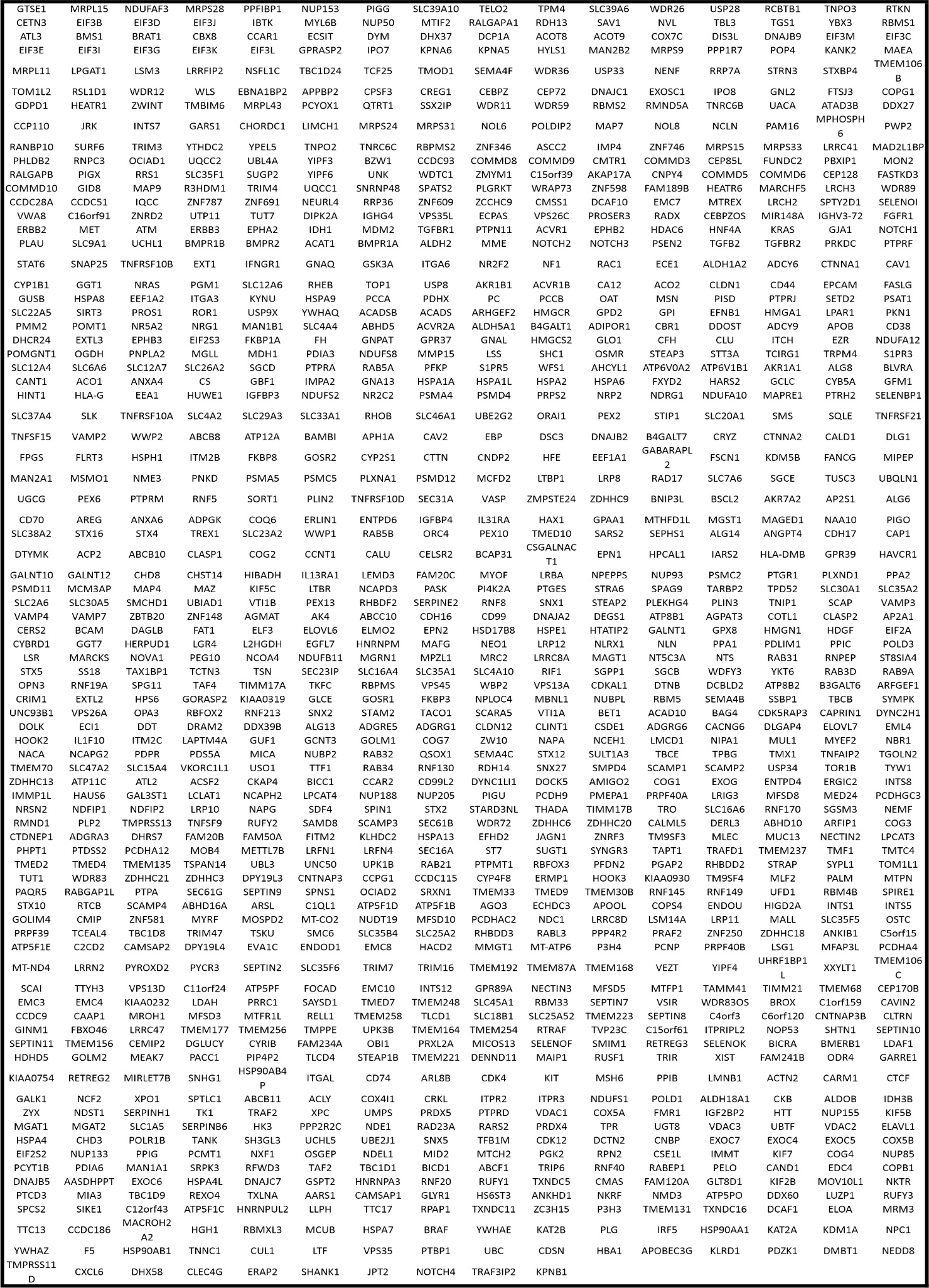


# 2.Supplementary Figures and Tables

## Supplementary Figures


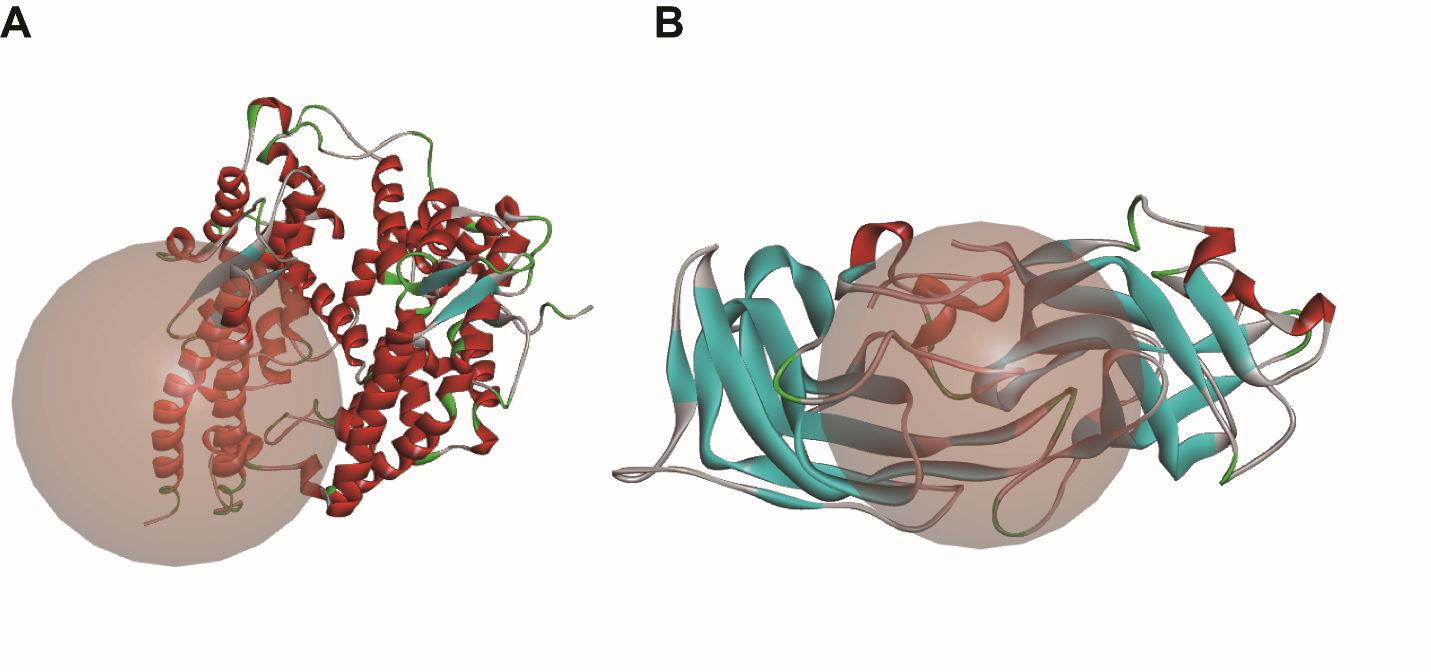


**Supplementary Figure 1.** (A) The defined binding region of ACE2. The sphere represents the defined binding region. The center sites of the sphere were based on the interfaced sites of ACE2 and SARS-CoV-2 S1. (B) The defined binding region of NRP1. The sphere represents the defined binding region. The center sites of the sphere were based on the interfaced sites of NRP1 and SARS-CoV-2 S1.
